# A GIS – based method for assessment and mapping of noise pollution in Ota metropolis, Nigeria

**DOI:** 10.1016/j.mex.2019.02.027

**Published:** 2019-02-27

**Authors:** S.O. Oyedepo, G.A. Adeyemi, O.C. Olawole, O.I. Ohijeagbon, O.K. Fagbemi, R. Solomon, S.O. Ongbali, O.P. Babalola, J.O. Dirisu, U.K. Efemwenkiekie, T. Adekeye, C.N. Nwaokocha

**Affiliations:** a Department of Mechanical Engineering, Covenant University, Nigeria; b Depatment of Civil Engineering, Covenant University, Nigeria; c Department of Physics, Covenant University, Nigeria; d Department of Mechanical Engineering, University of Ilorin, Nigeria; e Department of Mechanical Engineering, Olabisi Onabanjo University, Ibogun Campus, Nigeria

**Keywords:** A GIS – based method for developing noise map, Noise descriptors, Noise pollution level, Noise map, GPS, Background noise, Peak noise

## Abstract

A detailed method used for assessing and mapping noise pollution levels in Ota metropolis, Nigeria using ArcGIS 10.5 Software is presented in this paper. Noise readings were measured at a time interval of 30 min for each site considered using a precision grade sound level meter. The noise map developed was based on the computed values of average equivalent noise (L_Aeq_) for the selected locations. Results of this study show that the A weighted sound level (L_Aeq_), the background noise level (L_10_) and the peak noise level (L_90_) vary with location and period of the day due to traffic characteristics especially traffic volume, vehicle horns, vehicle mounted speakers, and unmuffled vehicles at road Junctions, major roads, motor parks and commercial centres. Based on the U.S. Department of Housing and Urban Development (HUD) recommendations and standards, only one (1) out of the 41 locations considered is under normally acceptable situation, while 12 locations are under normally unacceptable and the noise levels of the rest locations are clearly unacceptable. Results of this study are useful as reference and guideline for future planning and regulations on noise limit to be implemented for urban areas like Ota Metropolis.

• Instrumentation used in this study for the environmental noise measurements consisted of a precision-grade sound-level meter – Model 8922 RS232.
• The *Geographical Positioning System* (*GPS*) *device* (*model: Magellan eXplorist 310*) was used to obtain the exact coordinates of each location where noise level readings were recorded.
• ArcGIS 10.5 software was used in this study to develop noise map for Ota Metropolis.

## Graphical abstract

**Figure:**
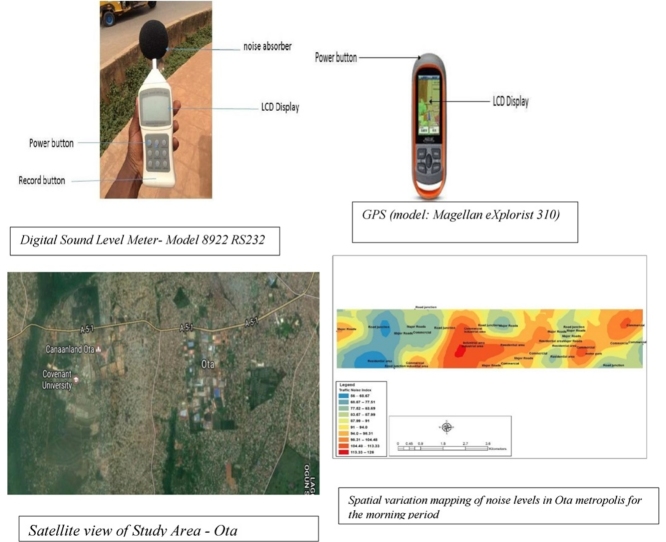

**Specifications Table**

**Table:** 

**Subject Area:**	Engineering
**More Specific Subject area:**	Mechanical Engineering, Environmental Engineering, Environmental Noise Control
**Method Name:**	A GIS – Based Method for Developing Noise Map
**Name and reference of original method:**	ArcGIS software – Esri: GIS Mapping Software
**Resource Availability:**	ArcGIS Software

## Method details

### Experimental procedures

Noise pollution is a significant environmental problem in many rapidly urbanizing areas [1,2]. In developing countries, like Nigeria, the problem of noise pollution is wide spread. There is no legal frame work upon which noise pollution can be abated. The high level of environmental noise reduces the quality of living [3] and the problem has to be tackled but first analyzed which is done by measuring the noise pollution level.

Instrumentation used in this study for the environmental noise measurements consisted of a precision-grade sound-level meter (according to IEC 651, ANSI S1.4 type 2 class standard) one 1/2-in. condenser microphone and 0.33-octave filter with frequency range and measuring level range of 31.5 Hz–8 kHz and 35–130 dB, respectively. The instruments were calibrated by the internal sound-level calibrator before making measurements at each selected site. The measurements were taken at street level (at road junctions, market centres, passengers loading parks, and residential areas). The instrument was held comfortably in hand with the microphone pointed at the suspected noise source at a distance above 1 m away from any reflecting object. *L*Ai (A-weighted instantaneous sound pressure level) measurements were recorded at intervals of 30 s for a period of 30 min, giving 60-meter readings per sampling location. This procedure was carried out for morning (7:00–9:00 a.m.), afternoon (2:00–4:00 p.m.) and evening (6:00–8:00 p.m.) measurements. A total of 41-locations were assessed for noise pollution level within Ota Metropolis [4]. From these readings, commonly used community noise descriptors such as the exceedence percentiles L_10_ and L_90_, the A-weighted equivalent sound pressure level, L_Aeq_, the daytime average sound level, L_D_, the noise pollution level, LNP, the traffic noise index, TNI, the noise climate (NC) and the noise exposure index (NEI) were computed.

These noise measures are defined as follows [5]:

(1) $$L_{Aeq}=10\text{log}10\;\frac{1}{N}\left[\sum_{i=1}^{i=k}\left(Antilog\frac{L_{Ai}}{10}ni\right)\right]$$

(2) $$LD=10\text{log}10\times \frac{1}{2}\left[Antilog\frac{L_{Aeq(m)}}{10}+Antilog\frac{L_{Aeq(A)}}{10}\right]$$

(3) $$LN=10\text{log}10\times \frac{1}{2}\left[Antilog\frac{L_{Aeq(E)}}{10}+Antilog\frac{L_{Aeq(N)}}{10}\right]$$

(4) TNI = 4 × (L_10_ – L_90_) + (L_90_ – 30) dB (A)

(5) LNP = L_eq_ + a (L_10_ – L_90_)

(6) *NC* = (*L_10_* – *L_90_*)

(7) *NEI* = (*t_1_/T_1_* + t_2_*/T_2_* + *…t_n_/T_n_*)

The noise descriptors for the selected locations at respective time of the day are presented in Table 1, Table 2, Table 3. While noise measurements were carried out and recorded, proper counting and recording of number of cars, tricycles, motorcycles and trucks that pass point of measurement were made at the selected locations close to the road. Also, the prevailing environmental condition was noted so as to know the major sources of the environmental noise in the surrounding. The sampling locations for the noise pollution monitoring were divided into zones/areas based on the predominant infrastructure or based on the notable characteristics of the area. The Geographical Positioning System (GPS) points were also collected for each location for accurate coordinates of the sampling points for the purpose of noise mapping.

**Table 1:** Minimum (Lmin), Maximum (Lmax) and percentile noise exceeded (L10, L50, L90) at Selected Locations.

Location	L_min_ dB(A)			L_max_ dB(A)			L_10_ dB(A)			L_50 dB_(A)			L_90_ dB(A)		
	M	A	E	M	A	E	M	A	E	M	A	E	M	A	E
Sifor Area	66	65	61	82	86	82	72	88	76	71	75	70	66	68	63
Bells University Junction	63	66	69	78	89	92	77	84	80	73	74	75	69	69	72
Canaan Land	65	65	63	90	101	85	80	88	78	72	75	72	67	69	67
May And Baker Close	45	43	31	67	65	63	56	56	55	48	49	45	46	45	41
High Court Area	63	63	65	86	89	83	82	79	80	77	75	76	72	70	70
Nestle	68	64	66	88	82	97	80	78	80	75	74	74	72	68	69
Iyana-Iyesi Market	59	60	77	81	78	87	74	72	82	66	67	79	63	62	78
Iyana-Iyesi Junction	69	69	72	95	86	89	84	83	85	78	75	78	73	71	74
Oju-Ore Junction	75	71	71	105	93	87	90	85	83	81	76	78	77	73	74
Joju Junction	63	63	67	89	84	84	79	79	79	74	73	73	68	69	69
Joju Express Road	73	72	73	88	94	94	86	88	82	80	80	77	75	75	75
Sango Under Bridge	73	75	73	102	113	110	91	96	87	79	83	78	75	77	75
Sango Car Park	60	55	67	92	89	94	80	80	84	68	71	73	61	71	68
Fowobi Junction	67	70	67	93	87	87	84	84	80	76	76	76	71	72	72
Toll Gate Express	67	65	71	85	98	88	86	76	85	74	71	78	70	67	73
Toll Gate Area	70	70	73	91	103	99	86	86	92	78	77	84	73	72	76
Obasanjo Junction	68	70	70	92	93	91	85	90	85	76	80	79	71	72	74
Ota-Market Area	66	68	67	85	94	94	82	84	83	75	77	77	70	72	71
Ogun State Internal Revenue	58	60	64	89	81	85	77	74	75	67	67	69	60	62	65
Ota Local Government Sct	63	63	63	91	87	82	77	75	77	71	70	72	66	65	67
Jack Ross Area (Road)	57	52	59	86	84	82	76	73	79	69	66	70	62	67	64
Chelsea (IDL)	55	57	69	99	83	90	80	78	87	72	71	82	63	63	75
Iganmode Sec School A/R	67	65	65	91	90	92	83	84	90	76	76	90	72	72	74
All-Over Polytechnic Road	61	64	75	84	95	94	82	84	90	76	74	81	65	66	76
Olota Palace Junction	66	65	69	90	86	86	80	78	80	74	73	78	70	68	73
Ijoko Road	66	59	53	90	84	95	82	81	85	74	77	79	70	68	71
Ijako Tipper Garrage	60	60	60	82	88	89	73	83	78	67	70	72	62	65	67
Ijoko Railway Station	59	64	53	91	82	81	81	80	78	70	75	72	62	69	65
Ilogbo Road	61	64	63	82	90	91	76	87	86	70	79	80	66	68	74
Ijoko Market	57	58	58	77	80	79	75	78	78	67	73	70	61	67	62
Ifo Road	68	66	68	93	88	87	86	83	83	80	79	78	71	72	70
Owode Area	64	65	56	88	82	85	80	80	80	74	78	76	68	68	66
Dalemo Junction	65	64	63	82	86	84	78	82	81	72	76	76	68	68	69
Ilo-Awela Road	60	62	66	84	83	84	74	81	82	68	73	77	63	66	71
Indomie	71	73	68	94	97	99	87	91	94	80	83	81	75	76	73
Tower Aluminum Company	51	50	48	79	72	71	75	71	68	59	64	59	55	55	53
Kolokote Area	55	51	52	87	73	74	81	61	70	62	59	63	56	54	56
Owode Area	64	69	64	92	91	90	89	86	88	78	80	79	73	75	73
Idiroko Road(Chelsea Area)	61	67	65	92	89	87	86	87	83	78	81	77	70	74	70
Bells University Drive	49	52	50	84	76	80	76	73	76	63	67	69	54	58	56
Estate	55	68	65	96	93	97	90	88	90	78	79	78	70	74	69

Key: M – Morning; A – Afternoon; E – Evening.

**Table 2:** Traffic noise Index (TNI), Pollution noise level (LNP) and Average Equivalent Noise Levels (LAeq) for the Selected Locations.

Location	TNI dB(A)			LNP dB(A)			L_Aeq_ dB(A)		
	M	A	E	M	A	E	M	A	E
Sifor Area	60	118	85	79.29	99.55	85.74	73.29	79.55	72.74
Bells University Junction	71	99	74	81.65	92.35	88.33	73.65	77.35	80.33
Canaan Land	89	115	81	90.65	106.09	86.06	77.65	87.09	75.06
May And Baker Close	56	59	67	62.68	64.42	65.14	52.68	53.42	51.14
High Court Area	82	76	80	88.52	85.82	86.71	78.52	76.82	76.71
Nestle Area	74	78	83	85.36	85.32	91.95	77.36	75.32	80.95
Iyana-Iyesi Market	77	72	64	81.83	78.93	83.63	70.83	68.93	79.63
Iyana-Iyesi Junction	87	89	88	92.44	90.18	91.89	81.44	78.18	80.89
Oju-Ore Junction	99	91	80	102.18	93.80	88.59	89.18	81.80	79.59
Joju Junction	82	79	79	87.79	85.20	85.13	76.79	75.20	75.13
Joju Express Road	89	97	73	93.16	96.62	87.73	82.16	83.62	80.73
Sango Under Bridge	109	123	93	103.92	115.57	105.36	87.92	96.57	93.36
Sango Car Park	107	77	102	95.82	85.80	96.73	76.82	76.80	80.73
Fowobi Junction	93	90	74	93.88	90.69	85.48	80.88	78.69	77.48
Toll Gate Express	104	73	91	93.14	92.11	91.95	77.14	83.11	79.95
Toll Gate Area	95	98	110	95.10	100.98	104.39	82.10	86.98	88.39
Obasanjo Junction	97	114	88	94.68	103.59	93.20	80.68	85.59	82.20
Ota-Market Area	88	90	89	90.01	93.03	92.98	78.01	81.03	80.98
Ogun State Internal Revenue Area	98	80	75	92.51	82.01	82.50	75.51	70.01	72.50
Ota Local Government Secretariat	80	75	77	87.10	83.30	84.04	76.10	73.30	74.04
Jack Ross Area (Road)	88	61	94	87.28	78.78	88.31	73.28	72.78	73.31
Chelsea (IDL)	101	93	93	99.43	88.22	95.15	82.43	73.22	83.15
Iganmode Sec School Area/Road	86	90	108	91.27	91.82	100.52	80.27	79.82	84.52
All-Over Polytechnic Road	103	108	102	94.53	98.73	99.43	77.53	80.73	85.43
Olota Palace Junction	80	78	71	88.04	85.59	85.30	78.04	75.59	78.30
Ijoko Road	88	90	97	90.64	91.03	96.06	78.64	78.03	82.06
Ijako Tipper Garrage	76	107	81	81.13	94.91	87.72	70.13	76.91	76.72
Ijoko Railway Station	108	83	87	97.05	87.12	86.89	78.05	76.12	73.89
Ilogbo Road	76	114	92	82.45	101.60	94.49	72.45	82.60	82.49
Ijoko Market	87	81	96	83.70	85.18	88.62	69.70	74.18	72.62
Ifo Road	101	86	92	97.85	91.01	92.40	82.85	80.01	79.40
Igbala	86	86	92	89.48	88.48	91.11	77.48	76.48	77.11
Dalemo Junction	78	94	87	84.22	91.93	89.62	74.22	77.93	77.62
Ilo-Awela Road	77	96	85	83.01	91.64	89.41	72.01	76.64	78.41
Indomie Area	93	106	127	95.34	101.67	109.22	83.34	86.67	88.22
Tower Aluminum Company	105	89	83	88.59	82.05	78.66	68.59	66.05	63.66
Kolokote Area	126	52	82	100.30	71.10	80.29	75.30	64.10	66.29
Owode Area	107	89	103	99.61	93.88	98.21	83.61	82.88	83.21
Idiroko Road(Chelsea Area)	104	96	92	98.07	95.21	92.26	82.07	82.21	79.26
Bells Drive	112	88	106	93.56	83.48	91.48	71.56	68.48	71.48
Estate	120	100	123	104.57	97.62	106.85	84.57	83.62	85.85

Key: M – Morning; A – Afternoon; E – Evening.

**Table 3:** Noise Exposure Index (NEI), Noise Climate (NC), LDay and Lnight for the Selected Locations.

Location	NEI			Noise Climate			LDay	LNight
	M	A	E	M	A	E	(L_D_)	(L_N_)
Sifor Area	1.04	1.14	1.04	6	20	13	77.5	72.7
Bells University Junction	1.05	1.11	1.15	8	15	8	75.9	80.3
Canaan Land	1.11	1.24	1.07	13	19	11	84.6	75.1
May And Baker Close	0.96	0.97	0.93	10	11	14	53.1	51.1
High Court Area	1.12	1.12	1.10	10	9	10	77.6	76.7
Nestle Area	1.05	1.08	1.16	8	10	11	76.5	81.0
Iyana-Iyesi Market	1.09	1.06	1.45	11	10	4	70.0	79.6
Iyana-Iyesi Junction	1.16	1.12	1.16	11	12	11	80.1	80.9
Oju-Ore Junction	1.37	1.26	1.45	13	12	9	86.9	79.6
Joju Junction	1.10	1.07	1.07	11	10	10	76.1	75.1
Joju Express Road	1.17	1.20	1.11	11	13	7	83.0	80.7
Sango Under Bridge	1.35	1.49	1.70	16	19	12	94.1	93.4
Sango Car Park	1.10	1.10	1.15	19	9	16	76.8	80.7
Fowobi Junction	1.16	1.12	1.11	13	12	8	79.9	77.5
Toll Gate Express	1.10	1.19	1.14	16	9	12	81.1	80.0
Toll Gate Area	1.26	1.34	1.61	13	14	16	85.2	88.4
Obasanjo Junction	1.15	1.22	1.17	14	18	11	83.8	82.2
Ota-Market Area	1.20	1.25	1.47	12	12	12	79.8	81.0
Ogun State Internal Revenue Ar	1.37	1.27	1.61	17	12	10	73.6	72.5
Ota Local Government Sect	1.38	1.33	1.65	11	10	10	74.9	74.0
Jack Ross Area (Road)	1.09	1.06	1.45	14	6	15	73.0	73.3
Chelsea (IDL)	1.10	0.98	1.28	17	15	12	79.9	83.2
Iganmode Sec School A/R	1.15	1.14	1.21	11	12	16	80.1	84.5
All-Over Polytechnic Road	1.11	1.14	1.22	17	18	14	79.4	85.4
Olota Palace Junction	1.20	1.16	1.42	10	10	7	77.0	78.3
Ijoko Road	1.12	1.11	1.17	12	13	14	78.4	82.1
Ijako Tipper Garrage	1.00	1.10	1.10	11	18	11	74.7	76.7
Ijoko Railway Station	1.20	1.17	1.34	19	11	13	77.2	73.9
Ilogbo Road	1.04	1.18	1.19	10	19	12	80.0	82.5
Ijoko Market	1.07	1.14	1.32	14	11	16	72.5	72.6
Ifo Road	1.18	1.14	1.13	15	11	13	81.7	79.4
Igbala	1.11	1.10	1.10	12	12	14	77.0	77.1
Dalemo Junction	1.06	1.11	1.11	10	14	12	76.5	77.6
Ilo-Awela Road	1.03	1.10	1.12	11	15	11	74.9	78.4
Indomie Area	1.19	1.24	1.26	12	15	21	85.3	88.2
Tower Aluminum Company	0.91	0.88	0.98	20	16	15	67.5	63.7
Kolokote Area	1.00	0.85	1.02	25	7	14	72.6	66.3
Owode Area	1.29	1.28	1.51	16	11	15	83.3	83.2
Idiroko Road(Chelsea Area)	1.17	1.17	1.13	16	13	13	82.1	79.3
Bells University Drive	1.10	1.05	1.30	22	15	20	70.3	71.5
Estate	1.13	1.11	1.32	20	14	21	84.1	85.9

### Geographical positioning system (GPS)

The *Geographical Positioning System* (*GPS*) *device* (*model: Magellan eXplorist 310*) was used to obtain the exact coordinates of each location where noise level readings were recorded. The GPS was turned on at each location and the latitude, longitude and altitude readings were taken. GPS readings were taken where there is no signal obstruction.

The GPS system currently has 31 active satellites in orbits inclined 55° to the equator. The satellites orbit about 20,000 km from the earth's surface The GPS receiver gets a signal from each GPS satellite. The satellites transmit the exact time the signals are sent. By subtracting the time the signal was transmitted from the time it was received, it can tell how far it is from each satellite and its receiver can determine the location of study in three dimensions which are east, north and altitude.

The eXplorist 310 version used in this study supports paperless geocaching and allows the use of more than 20 unique characteristics of each cache, including name, location, description, terrain, habitat and other details. The GPS is pre-loaded with the World Edition map. This unique preloaded map also includes geographical features (water features, urban and rural land use, and city centers).

Table A1 shows the geographical positioning systems coordinates while Table A2 shows the adopted codes for the selected 41 locations in Ota Metropolis.

### Noise mapping

Noise mapping as a graphical representation of the sound level distribution existing in a given region, it is an efficient noise assessment method in urban areas. It also helps in visualization of the noise distributions in areas where land uses are very sensitive to noise. This is one of the modern ways to assess noise levels and it helps in planning to mitigate noise pollution effects [6,7].

**Fig. 1:**
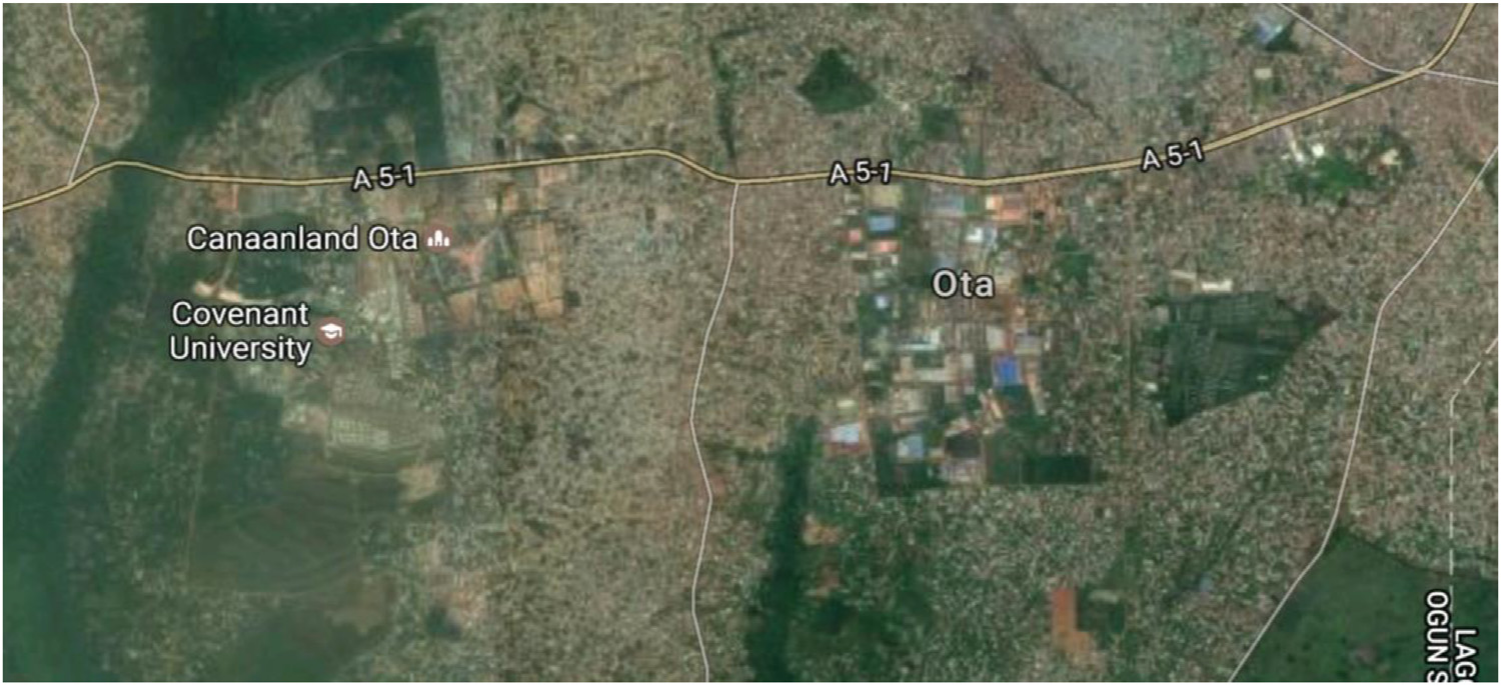
Satellite view of Ota (Image source: google Earth).

According to the Directive 2002/49/EC of the European Parliament and of the Council, of 25 June 2002 relating to the assessment and management of environmental noise imposes to its Member States the elaboration of noise maps for cities with more than 250,000 inhabitants, this was due on 30 June 2007 [1,2,8]. Based on this directive, Ota metropolis with population of over 527,242 inhabitants is due to be presented with noise map. Fig. 1 shows the satellite view of Ota.

In this study, development of noise map using GIS for selected noisy areas (commercial centers, major road junctions, passenger loading parks, high-density residential areas) and low-noise areas (low density residential areas) are presented. The data collected at the 41 locations were used to develop a noise map for the study location - Ota metropolis. Ota is one of major cities in South-West Nigeria. It is located between latitude 6° 38′N to 6° 41′N and longitude 03° 8′E and 3° 12′E. Fig. 4 shows the satellite view of the study area.

ArcGIS 10.5 software was used in this study to develop noise map for Ota Metropolis. The Software makes use of Inverse Distance Weighting (IDW) interpolation method. IDW provides satisfactory results when the number of elevation points in an area is large and the points are uniformly distributed. Also, the known sample points are implicit to be self-governing from each other [[9], [10], [11]]. Generally, interpolation helps to predict the cell values in a pattern format using a given number of sample data. It is a good tool for prediction of unknown values for a given geographic point data which in this study is noise.

**Fig. 2:**
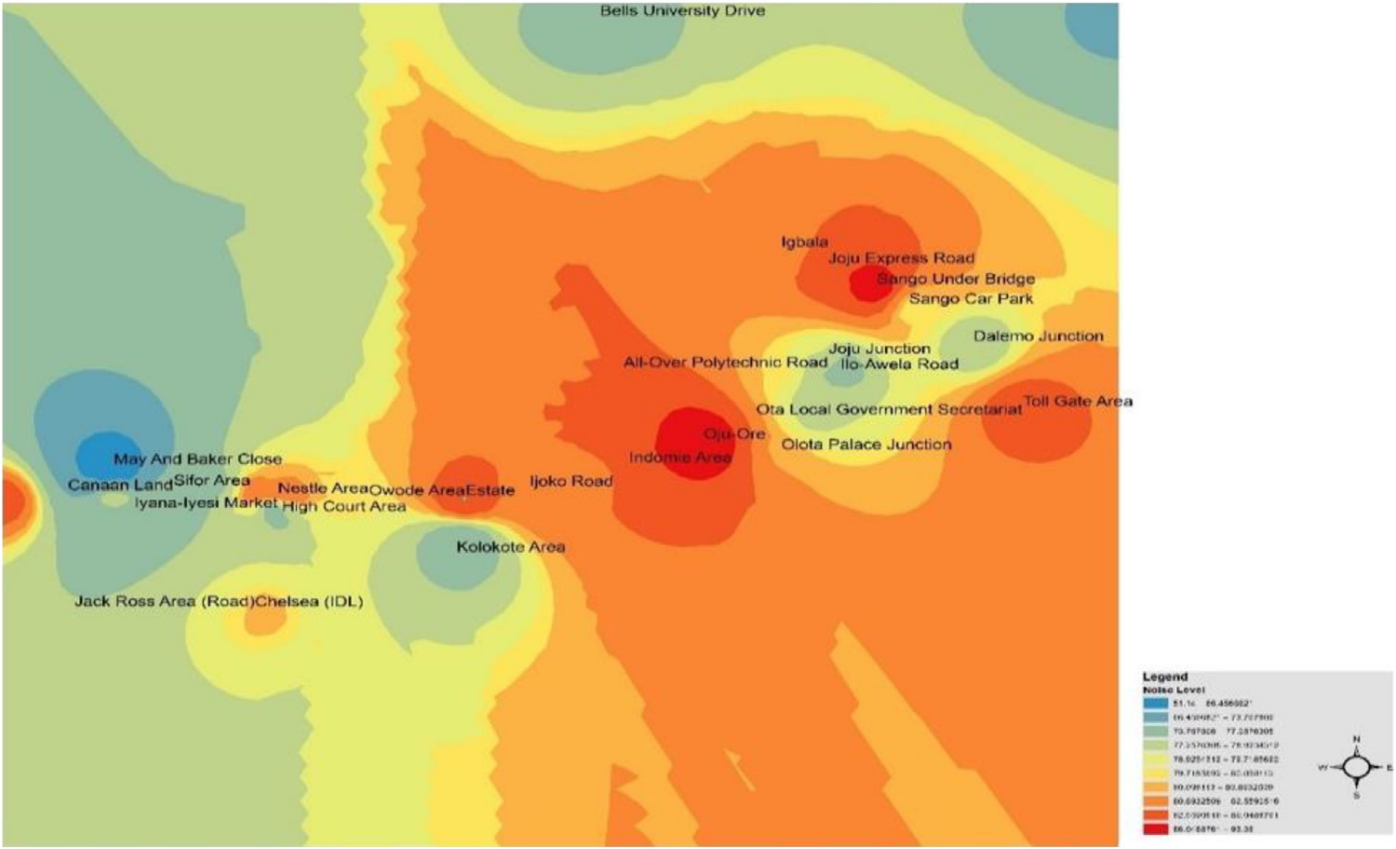
Spatial variation mapping of noise levels in Ota metropolis for the morning period.

**Fig. 3:**
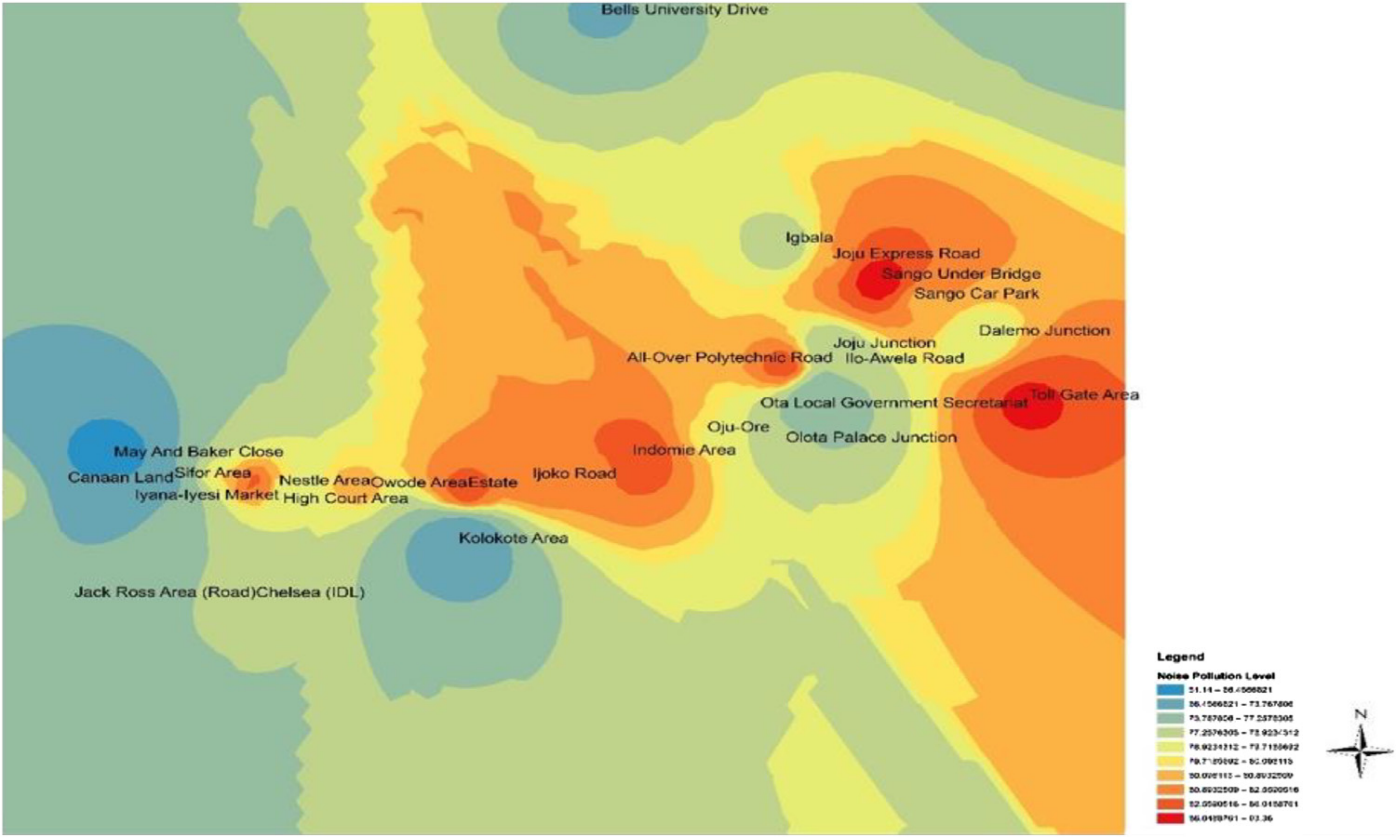
Spatial variation mapping of noise levels in Ota metropolis for the afternoon period.

**Fig. 4:**
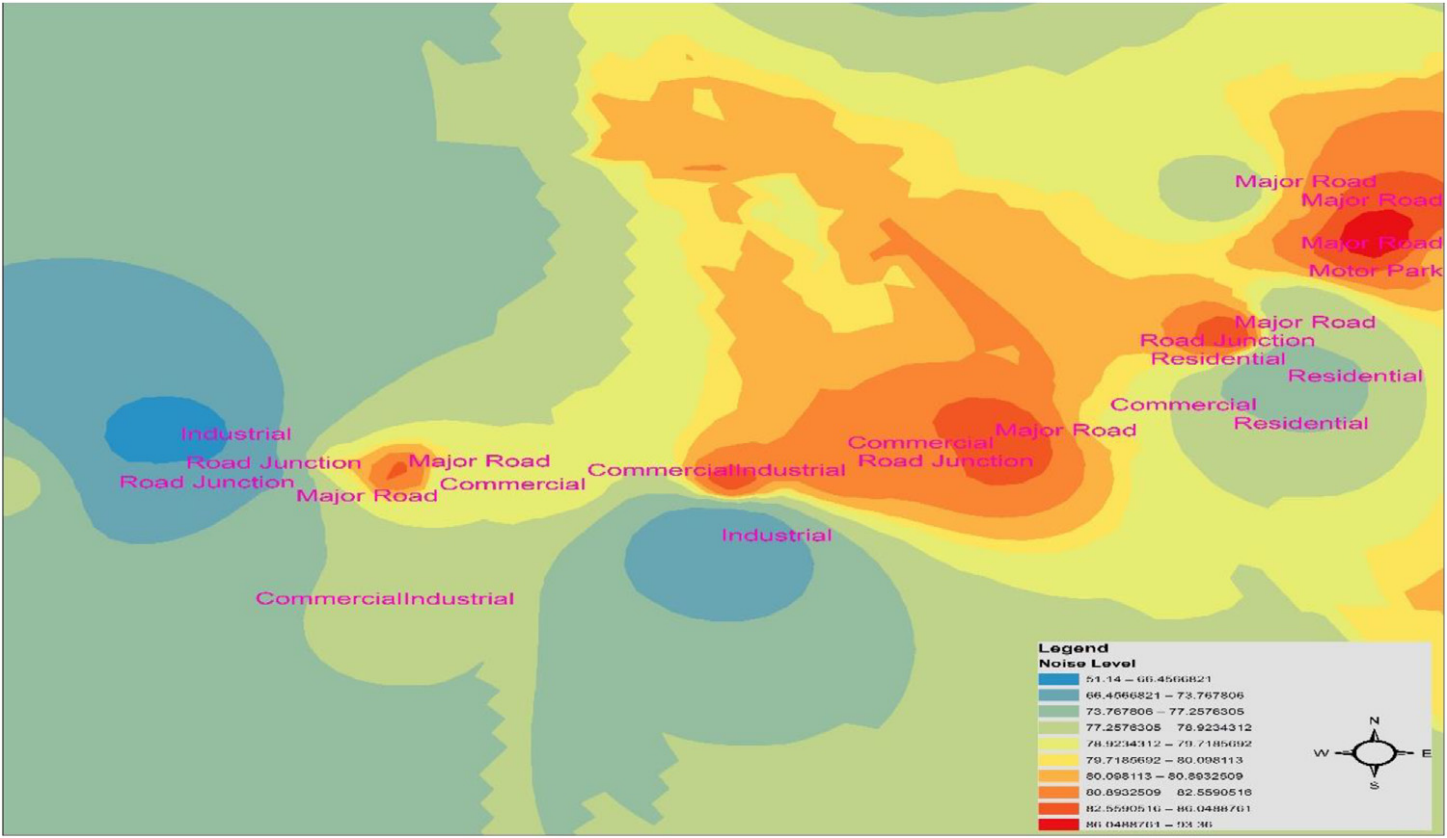
Spatial variation mapping of noise levels in Ota metropolis in the evening period.

The IDW Interpolation method is used by taking into consideration the data obtained from noise sources and distances between them. For its prediction, IDW utilises the given values surrounding the predicted location. It predicts that each given point has a local influence that shrinks with space; thereby giving greater weights to points closest to the prediction location, based on distance decay effect. This process leads to the procedure being referred to as inverse distance weighted. This technique was applied to measure the spatial distribution and range of acoustics in the area for the three periods of the day. Fig. 2, Fig. 3, Fig. 4 show the spatial variation mapping of noise levels in Ota metropolis for the morning, afternoon and evening periods of the day, respectively.

Results of this study show that the A weighted sound level (L_Aeq_), the background noise level (L_10_) and the peak noise level (L_90_) vary with location and period of the day due to traffic characteristics especially traffic volume, vehicle horns, vehicle mounted speakers, and unmuffled vehicles at road Junctions, major roads, motor parks and commercial centres. Based on the U.S. Department of Housing and Urban Development (HUD) recommendations and standards, only one (1) out of the 41 locations considered is under normally acceptable situation, while 12 locations are under normally unacceptable and the noise levels of the rest locations are clearly unacceptable.

## Figures and Tables

**Table A1:** Latitude and Longitude Coordinates for the Locations.

S/N	Location	Latitude	Longitude	Elevation
1	Sifor Area	6° 40′ 57.8′′	3° 10′ 24.3′′	75m
2	Bells University Junction	6° 41′ 00.2′′	3° 10′ 38.2′′	63m
3	Canaan Land	6° 40′ 55.7′′	3° 10′ 03.7′′	63m
4	May And Baker Close	6° 41′ 07.2′′	3° 10′ 03.1′′	62m
5	High Court Area	6° 40′ 52.7′′	3° 11′ 02.7′′	55m
6	Nestle Area	6° 40′ 54.4′′	3° 11′ 29.2′′	52m
7	Iyana-Iyesi Market	6° 40′ 48.0′′	3° 11′ 01.8′′	64m
8	Iyana-Iyesi Junction	6° 40′ 83.9′′	3° 11′ 04.9′′	65m
9	Oju-Ore	6° 41′ 18.2′′	3° 13′ 32.3′′	73m
10	Joju Junction	6° 41′ 55.8′′	3° 14′ 16.7′′	77m
11	Joju Express Road	6° 42′ 35.6′′	3° 14′ 16.5′′	78m
12	Sango Under Bridge	6° 42′ 26.6′′	3° 14′ 33.7′′	85m
13	Sango Car Park	6° 42′ 17.8′′	3° 14′ 45.2′′	82m
14	Fowobi Junction	6° 41′ 11.3′′	3° 13′ 13.0′′	81m
15	Toll Gate Express	6° 42′ 19.3′′	3° 14′ 47.2′′	80m
16	Toll Gate Area	6° 41′ 32.6′′	3° 15′ 25.6′′	80m
17	Obasanjo Junction	6° 40′ 58.2′′	3° 12′ 35.0′′	68m
18	Ota-Market Area	6° 41′ 03.8′′	3° 12′ 55.7′′	68m
19	Ogun State Internal Revenue Area	6° 41′ 35.6′′	3° 14′ 12.2′′	80m
20	Ota Local Government Secretariat	6° 41′ 29.0′′	3° 14′ 12.1′′	72m
21	Jack Ross Area (Road)	6° 40′ 04.6′′	3° 10′ 52.6′′	53m
22	Chelsea (IDL)	6° 40′ 04.4′′	3° 10′ 53.2′′	67m
23	Iganmode Sec School Area/Road	6° 40′ 56.3′′	3° 10′ 53.8′′	88m
24	All-Over Polytechnic Road	6° 41′ 49.2′′	3° 13′ 59.8′′	84m
25	Olota Palace Junction	6° 41′ 13.6′′	3° 13′ 59.9′′	78m
26	Ijoko Road	6° 40′ 57.4′′	3° 12′ 30.7′′	68m
27	Ijako Tipper Garrage	6° 44′ 34.3′′	3° 15′ 59.9′′	90m
28	Ijoko Railway Station	6° 44′ 58.0′′	3° 15′ 38.4′′	71m
29	Ilogbo Road	6° 44′ 57.4′′	3° 12′ 53.4′′	46m
30	Ijoko Market	6° 44′ 34.2′′	3° 15′ 60.0′′	92m
31	Ifo Road	6° 45′ 00.9′′	3° 12′ 53.0′′	47m
32	Igbala	6° 42′ 42.7′′	3° 13′ 75.0′′	69m
33	Dalemo Junction	6° 42′ 01.3′′	3° 15′ 08.1′′	60m
34	Ilo-Awela Road	6° 41′ 50.7′′	3° 14′ 20.9′′	83m
35	Indomie Area	6° 41′ 08.0′′	3° 13′ 05.9′′	78m
36	Tower Aluminum Company	6° 40′ 33.2′′	3° 12′ 06.5′′	67m
37	Kolokote Area	6° 40′ 28.5′′	3° 12′ 04.8′′	74m
38	Owode Area	6° 40′ 53.4′′	3° 12′ 07.9′′	69m
39	Idiroko Road(Chelsea Area)	6° 40′ 52.7′′	3° 09′ 23.7′′	64m
40	Bells Drive	6° 44′ 30.0′′	3° 12′ 55.0′′	51m
41	Estate	6° 40′ 53.4′′	3° 12′ 07.9′′	71m

**Table A2:** Codes Adopted for the Selected Locations.

S/N	LOCATION	CODE	PERIOD OF THE DAY	TIME OF THE DAY	DAY
1	SIFOR AREA	T_A1_	Morning	7:00–7:30 AM	SATURDAY
			Afternoon	2:00–2:30 PM	
			Evening	6:00–8:00 PM	
2	BELLS UNIVERSITY JUNCTION	T_A1_	Morning	7:30–8:00 AM	
			Afternoon	2:30–3:00 PM	
			Evening	6:00–8:00 PM	
3	CANAAN LAND	T_A1_	Morning	8:00–8:30 AM	
			Afternoon	3:00–3:30 PM	
			Evening	6:00–8:00 PM	
4	MAY AND BAKER CLOSE	B_A1_	Morning	8:30–9:00 AM	
			Afternoon	3:30–4:00 PM	
			Evening	6:00–8:00 PM	
5	HIGH COURT AREA	T_A2_	Morning	7:00–7:30 AM	SUNDAY
			Afternoon	2:00–2:30 PM	
			Evening	6:00–8:000 PM	
6	NESTLE AREA	T_A1_	Morning	7:30–8:00 AM	
			Afternoon	2:30–3:00 PM	
			Evening	6:00–8:00 PM	
7	IYANA-IYESI MARKET	C _A1_	Morning	8:00–8:30 AM	
			Afternoon	3:00–3:30 PM	
			Evening	6:00–8:00 PM	
8	IYANA-IYESI JUNCTION	T_A1_	Morning	8:30–9:00 AM	
			Afternoon	3:30–4:00 PM	
			Evening	6:00–8:00 PM	
9	OJU-ORE	C _A1_	Morning	7:00–7:30 AM	MONDAY
			Afternoon	2:00–2:30 PM	
			Evening	6:00–8:00 PM	
10	JOJU JUNCTION	T_A1_	Morning	7:30–8:00 AM	
			Afternoon	2:30–3:00 PM	
			Evening	6:00– 8:00 PM	
11	JOJU EXPRESS ROAD	T_A2_	Morning	8:00–8:30 AM	
			Afternoon	3:00– 3:30 PM	
			Evening	6:00–8:00 PM	
12	SANGO UNDER BRIDGE	C _A1_	Morning	8:30–9:00 AM	
			Afternoon	3:30–4:00 PM	
			Evening	6:00–8:00 PM	
13	SANGO CAR PARK	T_A3_	Morning	7:00–7:30 AM	TUESDAY
			Afternoon	2:00–2:30 PM	
			Evening	6:00–8:00 PM	
14	FOWOBI JUNCTION	T_A2_	Morning	7:30–8:00 AM	
			Afternoon	2:30–3:00 PM	
			Evening	6:00–8:00 PM	
15	TOLL GATE EXPRESS	T_A2_	Morning	8:00–8:30 AM	
			Afternoon	3:00–3:30 PM	
			Evening	6:00–8:00 PM	
16	TOLL GATE AREA	C _A1_	Morning	8:30–9:00 AM	
			Afternoon	3:30–4:00 PM	
			Evening	6:00–8:00 PM	
17	OBASANJO JUNCTION	T_A1_	Morning	7:00–7:30 AM	WEDNESDAY
			Afternoon	2:00–2:30 PM	
			Evening	6:00–8:00 PM	
18	OTA-MARKET AREA	C _A1_	Morning	7:30–8:00 AM	
			Afternoon	2:30–3:00 PM	
			Evening	6:00–8:00 PM	
19	OGUN STATE INTERNAL REVENUE AREA	R _A1_	Morning	8:00–8:30 AM	
			Afternoon	3:00–3:30 PM	
			Evening	6:00–8:00 PM	
20	OTA LOCAL GOVERNMENT SECRETARIAT	R _A1_	Morning	8:30–9:00 AM	
			Afternoon	3:30–4:00 PM	
			Evening	6:00–8:00 PM	
21	JACK ROSS AREA (ROAD)	C _A1_	Morning	7:00–7:30 AM	THURSDAY
			Afternoon	2:00–2:30 PM	
			Evening	6:00–8:00 PM	
22	CHELSEA (IDL)	B_A1_	Morning	7:30–8:00 AM	
			Afternoon	2:30–3:00 PM	
			Evening	6:00–8:00 PM	
23	IGANMODE SEC SCHOOL AREA/ROAD	T_A2_	Morning	8:00–8:30 AM	
			Afternoon	3:00–3:30 PM	
			Evening	6:00–8:00 PM	
24	ALL-OVER POLYTECHNIC ROAD	T_A2_	Morning	8:30–9:00 AM	
			Afternoon	3:30–4:00 PM	
			Evening	6:00–8:00 PM	
25	OLOTA PALACE JUNCTION	R _A1_	Morning	7:00–7:30 AM	FRIDAY
			Afternoon	2:00–2:30 PM	
			Evening	6:00–8:00 PM	
26	IJOKO ROAD	T_A2_	Morning	7:30–8:00 AM	
			Afternoon	2:30–3:00 PM	
			Evening	6:00–8:00 PM	
27	IJAKO (TIPPER GARRAGE)	T_A2_	Morning	8:00 –8:30 AM	
			Afternoon	3:00–3:30 PM	
			Evening	6:00–8:00 PM	
28	IJOKO RAILWAY STATION	C _A1_	Morning	8:30–9:00 AM	
			Afternoon	3:30–4:00 PM	
			Evening	6:00–8:00 PM	
29	ILOGBO ROAD	T_A2_	Morning	7:00–7:30 AM	SATURDAY
			Afternoon	2:00–2:30 PM	
			Evening	6:00–8:00 PM	
30	IJOKO MARKET	C _A1_	Morning	7:30–8:00 AM	
			Afternoon	2:30–3:00 PM	
			Evening	6:00–8:00 PM	
31	IFO ROAD	T_A2_	Morning	8:00–8:30 AM	
			Afternoon	3:00–3:30 PM	
			Evening	7:00–7:30 PM	
32	IGBALA	T_A2_	Morning	8:30–9:00 AM	
			Afternoon	3:30–4:00 PM	
			Evening	7:30–8:00 PM	
33	DALEMO JUNCTION	T_A1_	Morning	7:00–7:30 AM	SUNDAY
			Afternoon	2:00–2:30 PM	
			Evening	6:00–6:30 PM	
34	ILO-AWELA ROAD	T_A1_	Morning	7:30–8:00 AM	
			Afternoon	2:30–3:00 PM	
			Evening	6:00–8:00 PM	
35	INDOMIE AREA	T_A2_	Morning	8:00–8:30 AM	
			Afternoon	3:00–3:30 PM	
			Evening	6:00–8:00 PM	
36	TOWER ALUMINUM COMPANY	B_A1_	Morning	8:30–9:00 AM	
			Afternoon	3:30–4:00 PM	
			Evening	6:00– 8:00 PM	
37	KOLOKOTE AREA	B_A1_	Morning	7:00–7:30 AM	MONDAY
			Afternoon	2:00–2:30 PM	
			Evening	6:00–8:00 PM	
38	OWODE AREA	C _A1_	Morning	7:30–8:00 AM	
			Afternoon	2:30–3:00 PM	
			Evening	6:00–8:00 PM	
39	IDIROKO ROAD (CHELSEA AREA)	T_A2_	Morning	8:00–8:30 AM	
			Afternoon	3:00–3:30 PM	
			Evening	6:00–8:00 PM	
40	BELLS DRIVE	R _A1_	Morning	8:30–9:00 AM	
			Afternoon	3:30–4:00 PM	
			Evening	7:30–8:00 PM	
41	ESTATE AREA	B_A1_	Morning	7:00–7:30 AM	TUESDAY
			Afternoon	2:00–2:30 PM	
			Evening	6:00–8:00 PM	

Key: C _A1_ – Commercial Centres ; T_A1_ – Road traffic area (Road junction); T_A2_ – Road traffic area (Major Road); B_A1_ – Industrial area; R _A1_ – Residential area.
